# Supplementary material for: Intermittent cell division dynamics in regenerating Arabidopsis roots reveals complex long-range interactions
Source: Quant Plant Biol. 2024 Sep 4;5:e7. doi: 10.1017/qpb.2024.7 (PMC11706681; doi:10.1017/qpb.2024.7)
Supplement: Fallesen et al. supplementary material [file S2632882824000079sup001.pdf]

Intermittent cell division dynamics in regenerating Arabidopsis roots reveals complex long-range interactions

T. Fallesen[1,2], S. Amarteifio[3], G. Pruessner[3], H. J. Jensen[3,4], G. Sena[1*]

[1]Department of Life Sciences, Imperial College London, UK.

[2]Crick Advanced Light Microscopy, Francis Crick Institute, London, UK.

[3]Department of Mathematics, Imperial College London, UK.

[4]Centre for Complexity Science , Imperial College London, UK

* Author for correspondence: g.sena@imperial.ac.uk

SUPPLEMENTARY FIGURES

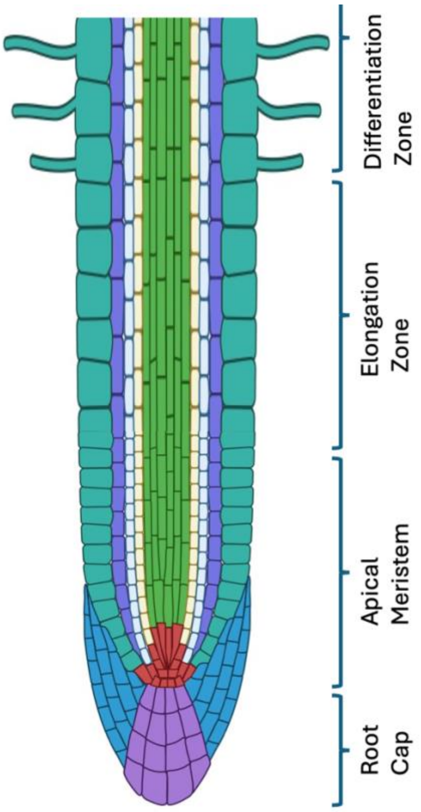

Supplementary Figure S1. Diagram of the *Arabidopsis* primary root tip. Cell proliferation occurs in the apical meristem, which is where the CYCB1;1::GFP signal was imaged in this work. In regenerating roots, the excision was performed right above the stem cell niche (red cells).

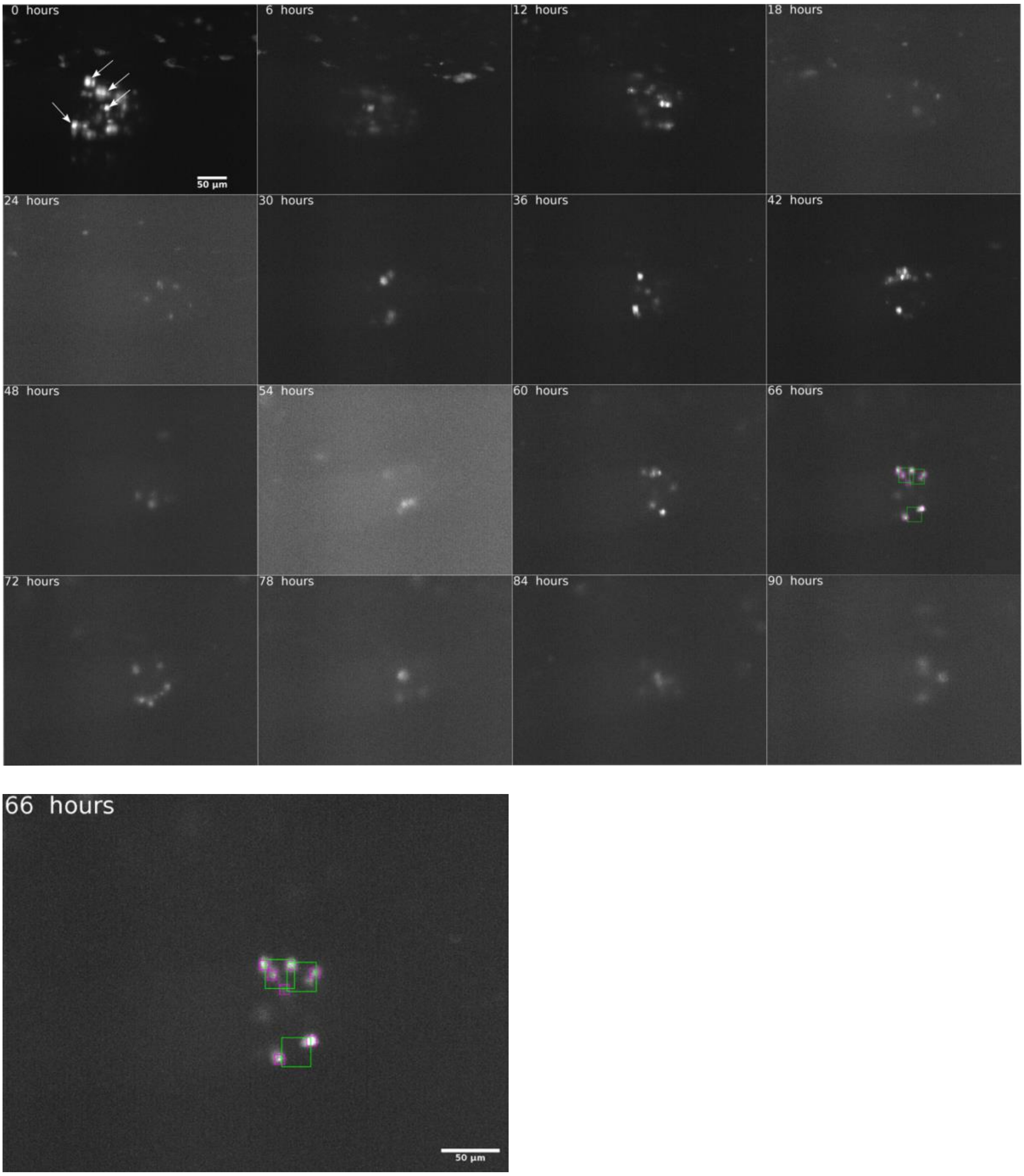

Supplementary Figure S2*.* (Top) Raw data with maximum intensity projections from time-lapse imaging of a representative regenerating primary root in *Arabidopsis* expressing CYCB1;1::GFP; the white blobs are GFP-positive cells, indicating mitotic events (arrows in the first panel point at some examples). (Bottom) Magnified version of the 66 hours frame, showing three clusters of events; Magenta, regions with 8.24 µm diagonal (see main text); Green, regions with 24.72 µm diagonal (see main text).
