# Supplementary material for: Intermittent cell division dynamics in regenerating Arabidopsis roots reveals complex long-range interactions
Source: Quant Plant Biol. 2024 Sep 4;5:e7. doi: 10.1017/qpb.2024.7 (PMC11706681; doi:10.1017/qpb.2024.7)
Supplement: Peer review history

# Editor report: Recommendation: Intermittent cell division dynamics in regenerating Arabidopsis roots reveals complex long-range interactions — R0/PR2

**DOI:** 10.1017/qpb.2024.7.pr2

**Affiliations:** School of Life Sciences, University of Warwick, United Kingdom of Great Britain and Northern Ireland

Dear Giovanni

Thanks for your submission to QPB. This has been reviewed by 2 experts in the field. We invite you to respond to their comments, in particular the inclusion of a reporter by reviewer 1.

Best wishes

George

# Editor report: Decision: Intermittent cell division dynamics in regenerating Arabidopsis roots reveals complex long-range interactions — R0/PR3

**DOI:** 10.1017/qpb.2024.7.pr3

**Affiliations:** RDP, ENS de Lyon, France

No accompanying comment.

# Editor report: Recommendation: Intermittent cell division dynamics in regenerating Arabidopsis roots reveals complex long-range interactions — R1/PR5

**DOI:** 10.1017/qpb.2024.7.pr5

**Affiliations:** School of Life Sciences, University of Warwick, United Kingdom of Great Britain and Northern Ireland

The authors have addressed all reviewer comments.

# Editor report: Decision: Intermittent cell division dynamics in regenerating Arabidopsis roots reveals complex long-range interactions — R1/PR6

**DOI:** 10.1017/qpb.2024.7.pr6

**Affiliations:** RDP, ENS de Lyon, France

No accompanying comment.
